# High-Energy Supplemental Feeding Shifts Gut Microbiota Composition and Function in Red Deer (*Cervus elaphus*)

**DOI:** 10.3390/ani14101428

**Published:** 2024-05-10

**Authors:** Peng Zheng, Weizhen Gao, Shaobo Cong, Lin Leng, Tao Wang, Lei Shi

**Affiliations:** 1 College of Animal Sciences, Xinjiang Agricultural University, Urumqi 830052, China; zp717lx@163.com (P.Z.); gao7222@yeah.net (W.G.); 2 Key Laboratory of Ecological Adaptation and Evolution of Extreme Environment Biology in Xinjiang, College of Life Sciences, Xinjiang Agricultural University, Urumqi 830052, China; ll121519as@126.com (L.L.); 13982544709@163.com (T.W.); 3 Xinjiang Tianshan Wildlife Park, Urumqi 830039, China; 13579236696@139.com

**Keywords:** supplemental feeding, gut microbiota, microbiological function, *Cervus elaphus*

## Abstract

**Simple Summary:**

Dietary selection affects the survival and health of mammals under winter, high-energy supplemental feeding (SF) is one of the commonly used strategies for captive wildlife, however, how gut microbiota respond to high-energy dietary in winter remains poorly understood. 16S rRNA gene analysis was employed to determine gut microbiota in red deer (*Cervus elaphus*) in winter. High-energy feed affects the gut microbial composition and function in red deer. During SF, the gut microbes in red deer were enriched in microorganisms associated with butyrate and lipid metabolism, such as *R. microfusus*, *M. intestinale*, and *Papillibacter cinnamivorans*. These gut microbes may be involved in ameliorating obesity associated with high-energy diets. Our findings indicate effectiveness of high-energy supplementary feeding.

**Abstract:**

Winter supplemental feeding (SF) is commonly used to improve the survival of captive wildlife. To investigate the impact of winter supplementation on the gut microbiota of wildlife, we assessed changes in the gut microbiota of red deer (*Cervus elaphus*) during the supplementary and non-supplementary feeding (NSF) groups using 16S rRNA sequencing technology. We found no significant differences in the diversity of the gut microbiota between SF and NSF except for the Simpson’s index. However, the relative abundance of Bacteroidetes, Lentisphaerae, and Proteobacteria in the gut microbiota was significantly higher during SF. Further, genera such as *Intestinimonas*, *Rikenella*, *Lawsonibacter*, *Muribaculum*, and *Papillibacter* were more abundant during SF. Beta diversity analysis showed significant differences between SF and NSF. The microbes detected during SF were primarily associated with lipid metabolism, whereas those detected during NSF were linked to fiber catabolism. High-energy feed affects the gut microbial composition and function in red deer. During SF, the gut microbes in red deer were enriched in microorganisms associated with butyrate and lipid metabolism, such as *R. microfusus*, *M. intestinale*, and *Papillibacter cinnamivorans*. These gut microbes may be involved in ameliorating obesity associated with high-energy diets. In summary, SF is a reasonable and effective management strategy.

## 1. Introduction

Gut microbes are closely associated with host health. Specific gut microbiota aid energy and nutrient absorption, vitamin synthesis, and detoxification of plant defense compounds in herbivorous mammals [1]. Biotic factors influencing the composition of the gut microbiota include feed composition, additives, genetics, and the physiological status (age and health) of the host [2,3,4,5,6]. Abiotic factors, including the season, habitat variation, and feeding schedules, also impact the gut microbiota [7,8,9]. In wildlife, the effects of season and environment are more pronounced. Several rare wildlife species are subjected to captive breeding, such as giant pandas (*Ailuropoda melanoleuca*) [10], snub-nosed monkeys (*Rhinopithecus roxellana*, *R. brelichi*, and *R. bieti*) [11], and black rhinoceros (*Diceros bicornis*) [12]. Maintaining the gut health of captive species is crucial for their survival. Further, investigating how seasonal changes, host–microbe co-evolution, and anthropogenic factors such as winter supplementation affect gut microbe diversity is vital for wildlife conservation and management and for understanding their potential to adapt to environmental changes.

Cervidae is the second-most abundant family of Artiodactyla, containing 46 species [13]. In Cervidae, *Cervus elaphus* is second only to the moose in size and was classified as a least concern on the 2019 IUCN Red List [14]. However, in China, it is a class II national protected animal. A few studies of gut microorganisms in the Artiodactyla family showed that the alpha diversity of the bacterial community is significantly higher in the summer than in the winter. This difference in the gut microbiota in mule deer and white-tailed deer is attributed to lower feed utilization [15]. Similarly, studies on white-lipped deer (*Cervus albirostris*) have revealed enrichment in various microbiota phyla, particularly Firmicutes, Bacteroidetes, and Patescibacteria, during the grassy season, whereas the abundance of Actinobacteria and Proteobacteria was increased during the dry grass season [16]. Interestingly, differences in the gut microbiota enrichment due to seasonal changes are often associated with an animal’s diet [17,18,19,20]. In captive wildlife such as Rocky Mountain elk (*Cervus canadensis nelsoni*), the composition of winter supplemental feeds may lead to changes in their gut microbiota [18]. For example, alfalfa pellet supplementation alters the composition of the bacterial microbiota compared to the addition of dry grasses [21]. Similarly, another study found higher gut microbiota diversity in Père David’s deer (*Elaphurus davidianus*) fed regular diets consisting of silage alone or natural vegetation compared with that following feeding with a mixture of silage and natural vegetation [22]. Similar results were observed in a gut microbiota study of captive *C. elaphus*, where the proportion of thick-walled bacteria phyla was correlated with silage consumption [23]. Notably, sex, diet, and environment influence changes in the gut microbiota [23,24,25].

The classical models of ungulate foraging [26,27,28] are premised on the strategy of energy maximization. Especially in winter, the nutritional requirements of wild ungulates are even more demanding because of the low availability of food resources. When food resources are limited, large herbivores will either consume high-energy foods in the short term [29,30,31] or increase the rate of food intake, e.g., red deer, Highland cattle, and Konik ponies all select food according to the principle of maximizing the intake rate, with the highest rate of intake (72.1%) in particular in red deer [32].

Winter supplemental feeding is a common practice to maintain captive wildlife populations, such as population reproductive rates in captive wildlife [33]. Although this approach keeps food resources available for deer during the winter, easily fermentable supplemental feeding promotes the proliferation of captive ruminant phenotypes associated with acidosis [33]. Similar adaptations may occur in domestic ruminants and negatively affect their health status [33]. Although previous studies have compared gut microbiota diversity between wild and captive species [23,25] and investigated feed formulation effects [34], few studies have focused on red deer in wildlife parks (supplemental feeding recipes are shown in Table S1 in winter and free-ranging in summer).

In this study, we evaluated the gut microbiota of captive red deer in the Xinjiang Tianshan Wildlife Park to examine the following three presumptions:

Presumption 1: The diversity of the gut microbiota is higher in the non-supplemented season than in the supplemented feeding season.

Presumption 2: Supplementation with high-energy feed affects the composition and function of the gut microbiota in red deer, leading to an increased abundance of pathogenic bacteria.

Presumption 3: High-energy supplementation feeds in the winter enrich energy and lipid metabolism-associated gut microbiota during the supplemental feeding season.

## 2. Materials and Methods

### 2.1. Study Area

The study area was Xinjiang Tianshan Wildlife Park (87.7866° N, 43.6688° E), which is at the southern foot of the Bogda Peak in the Tianshan Mountains, Daban City District, China, and covers an area of 75 km^2^. It is mainly composed of alluvial plains and mountains. Desert plants, such as *Artemisia* spp., *Bassia prostrata*, *Anabasis brevifolia*, *Nanophyton erinaceum*, *Ephedra sinica*, *Reaumuria songonica*, *Caragana sinica*, *Neotrinia splendens*, and *Phragmites australis*, grow on the plains that fan out in front of the mountains. In the subalpine mountainous areas, plants such as *Rosa* spp., *Caragana sinica*, *Lonicera japonica*, *Convolvulus fruticosus*, *Stipa capillata*, *Chrysanthemum indicum*, *Achnatherum inebrians*, and *Kali collinum* are found [35]. In mountainous areas, ungulates such as red deer, ibex (*Capra sibirica*), and argali (*Ovis ammon*) roam freely. These ungulates feed on natural vegetation in the summer and at fixed points in the winter. Supplementary feeding (SF) during the winter months is based on high-energy grains such as maize (see Table S1 for a detailed recipe).

### 2.2. Fecal Sample Collection

Fecal samples of *C. elaphus* were collected from high mountainous areas of the Xinjiang Tianshan Wildlife Park. During winter, we monitored the behavior of the deer using a clockwise scanning method, and when we observed the deer defecating, we immediately collected fresh fecal samples from that location in freezing tubes, which were preserved in 95% ethanol [36]. In summer, we tracked the deer groups using animal trails and footprints because deer feces (bullet-shaped pellets) are visually significantly different from those of the sympatric ibex (small, ball-shaped particles) and yak (large chunks) [37]. The degree of freshness was determined by the wetness of the feces and the adherence of mucous membranes to the epidermis [38], and only samples with visible mucous membranes on the epidermis and a soft texture were collected as fresh fecal samples. We collected fecal samples using sterile polyethylene gloves, and 10–15 fresh fecal pellets from each fresh fecal pile near the site where the deer were observed were taken as a single fresh fecal sample in multiple 10 mL sterile freezer tubes containing 95% ethanol. We collected 38 fresh fecal samples (18 during the SF and 20 during the non-supplementary feeding [NSF] groups) in the high mountainous areas of the Wildlife Park in early March 2023 and June 2023. Fresh fecal samples were stored at −20 °C in 95% ethanol solutions for cold storage and transport after collection [39]. To avoid individual differences, red deer feces were collected during the SF and NSF groups and mixed thoroughly in unused freezing tubes to obtain one 200 mg mixed sample each for the SF and NSF groups, which was used for subsequent analyses [40].

### 2.3. DNA Extraction and PCR Amplification

Microbial DNA was extracted from *C. elaphus* fecal samples using the E.Z.N.A.^®^ Soil DNA Kit (Omega Bio-tek, Norcross, GA, USA) following the manufacturer’s instructions. The V1–V9 region of the bacterial 16S ribosomal RNA gene was amplified using PCR (95 °C for 2 min, followed by 27 cycles at 95 °C for 30 s, 55 °C for 30 s, and 72 °C for 60 s, and final extension at 72 °C for 5 min) using primers 27F 5′-AGRGTTYGATYMTGGCTCAG-3′ and 1492R 5′-RGYTACCTTGTTACGACTT-3′ [41], where the barcode was an eight-base sequence unique to each sample. PCR was performed in triplicate with 20 μL mixtures containing 4 μL of 5 × FastPfu Buffer, 2 μL of 2.5 mM dNTPs, 0.8 μL of each primer (5 μM), 0.4 μL of FastPfu polymerase, and 10 ng of template DNA. Amplicons were extracted using 2% agarose gels and purified using an AxyPrep DNA Gel Extraction Kit (Axygen Biosciences, Union City, CA, USA) following the manufacturer’s instructions.

### 2.4. Library Construction and Sequencing

SMRTbell libraries (SMRTbell Prep Kit 3.0.) were prepared from the amplified DNA through blunt ligation following the manufacturer’s instructions (Pacific Biosciences, Menlo Park, CA, USA). Purified SMRTbell libraries from the Zymo (Irvine, CA, USA) and HMP mock communities were sequenced on dedicated PacBio Sequel cells using S/P1-C1.2 sequencing chemistry. Purified SMRTbell libraries from pooled and barcoded samples were sequenced on a single PacBio Sequel cell. Replicate 1 of the samples was sequenced using the S/P2-C2/5.0 sequencing chemistry and replicate 2 of the samples was sequenced using a pre-release version of S/P3-C3/5.0 sequencing chemistry. Amplicon sequencing was performed by Shanghai Biozeron Biotechnology Co., Ltd. (Shanghai, China).

### 2.5. Sequence Processing and Analysis

PacBio raw reads were processed using SMRT Link Analysis software version 9.0 to obtain demultiplexed circular consensus sequence reads with the following settings: minimum number of passes = 3, minimum predicted accuracy = 0.99. Raw reads were processed using the SMRT Portal (SMRTLINK v11) to filter sequences for length (1200–1800 bp) and quality. Sequences were further filtered by removing barcodes and primer sequences.

Operational taxonomic units (OTUs) that passed a 98.65% similarity cutoff were clustered using UPARSE (version 10; http://drive5.com/uparse/, accessed on 10 January 2024). The phylogenetic affiliation of each 16S rRNA gene sequence was analyzed using the UCLUST algorithm (https://github.com/topics/uclust, accessed 10 January 2024) against the Silva (SSU138.1) 16S rRNA database (http://www.arb-silva.de, accessed on 10 January 2024) with a confidence threshold of 80% [42].

### 2.6. Alpha and Beta-Diversity Analyses

Rarefaction analysis based on Mothur v.1.21.1 [43] was conducted to determine the diversity indices, including the Chao, ACE, and Shannon diversity indices. Beta diversity analysis was performed using UniFrac [44] to compare the results of the principal component analysis (PCA) using the community ecology package “R-forge” (the “vegan 2.0-0” package was used to generate a PCA figure). One-way analysis of variance (ANOVA) tests was employed to statistically assess the differences in diversity indices between samples. One-way permutational ANOVA and analysis of similarities (ANOSIM) were performed using the R “vegan” package to assess differences between the bacterial communities in the two groups [45,46].

### 2.7. Analysis of Species Composition and Function

The relative differential abundances of gut microbes at the phylum and genus levels were evaluated using a *t*-test. Differences were considered statistically significant at *p* < 0.05. We corrected for the *p*-value using the Benjamini–Hochberg method [47]. Similarity percentage difference contribution analysis was performed using the “simper” function in the “vegan” package to quantify the contribution of each species to the difference between the two groups. Overall variability between groups was calculated using ANOSIM [48]. Linear discriminant analysis (LDA) effect size (LEfSe) analysis was also conducted [49]. The Kruskal–Wallis sum-rank test was performed to examine the changes and dissimilarities among classes, followed by LDA to determine the effect size for each distinctively abundant taxa [50]. The Phylogenetic Investigation of Communities by Reconstruction of Unobserved States (PICRUSt2) (http://picrust.github.io/picrust/tutorials/genome_prediction.html, accessed on 10 January 2024) program, based on the Kyoto Encyclopedia of Genes and Genomes (KEGG) database, was used to predict functional alterations among microbiota in different samples. The obtained OTU data were used to generate BIOM files formatted as inputs for PICRUSt2 in BIOM script, which can be read in Mothur v.1.21.1 [51]. Functional pathways with significant differences were analyzed using STAMP 2.1.3 [52] Further intergroup differences in the relative abundances of KEGG levels 1, 2, and 3 pathways were compared using the Wilcoxon rank-sum test.

## 3. Results

### 3.1. Sequencing Information

**Figure 1:**
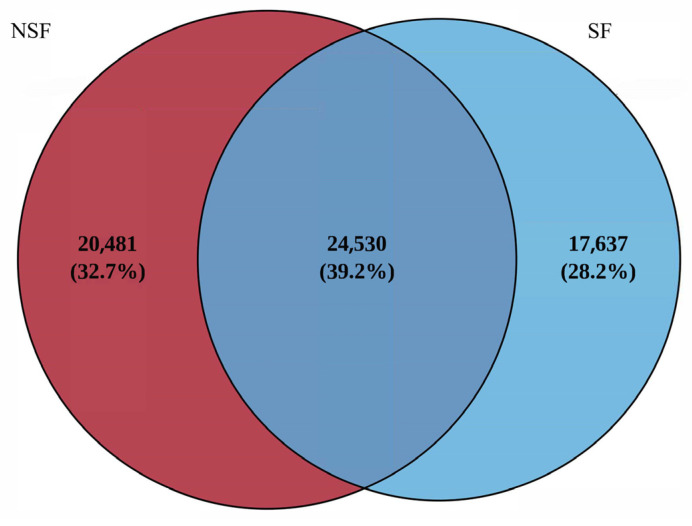
Shared and unique operational taxonomic units of red deer among the two groups are visualized using a Venn diagram. NSF, non-supplemental feeding; SF, supplemental feeding.

A total of 1,229,803 paired-end reads were generated from the 38 samples collected during the two groups. In total, 1,220,301 clean reads were obtained after quality control and assembly, and the two groups were clustered into 62,648 OTUs. The Venn diagram in Figure 1 shows that 24,530 OTUs were present in all 38 samples from both groups and consisted of sequences from Firmicutes (33.98%) and Bacteroidetes (53.40%). The core families identified were Oscillospiraceae, Eubacteriales, Lactobacillaceae, Lachnospiraceae, and Rikenellaceae. Library coverage of all samples was >99.23%, which indicated that the sequencing volume was sufficient to cover all samples (Figure S1).

### 3.2. Analysis of Gut Microbiota Diversity

**Figure 2:**
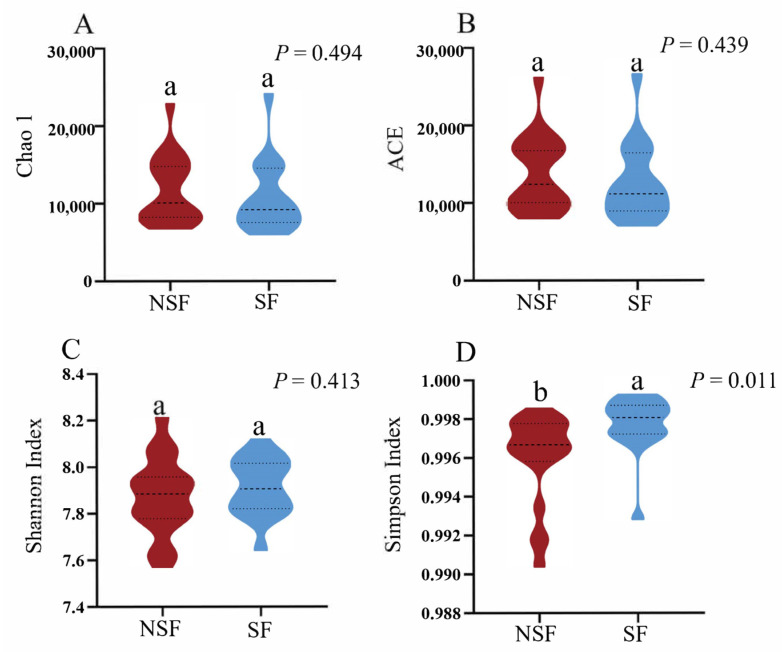
Differences in the alpha diversity of gut microbiota of red deer between the two groups. (**A**) Chao1; (**B**) ACE; (**C**) Shannon index; (**D**) Simpson index. Note: Different letters indicate significant differences at the *p* < 0.05 level.

Alpha diversity reveals the homogeneity and richness of the microbial composition between samples. The Chao1, ACE, and Shannon indices of the gut microbiota did not differ between the two groups (Figure 2A–C). However, the Simpson index of the gut microbiota was significantly higher in red deer during SF than during NSF (Figure 2D).

**Figure 3:**
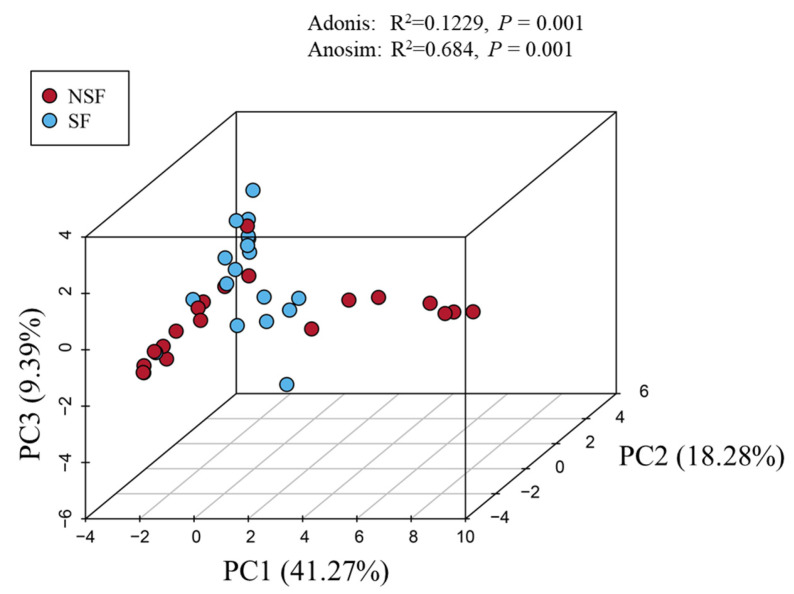
Beta diversity of the gut microbiota of red deer between the two groups.

The PCA plot indicated the distance between the microbial communities in the two composite samples (Figure 3). Notably, microbiota compositions were significantly different in the two groups. This difference was confirmed through permutational ANOVA (R^2^ = 0.1229, *p* = 0.001) and ANOSIM (R^2^ = 0.684, *p* = 0.001).

### 3.3. Gut Microbiota Community Composition

**Figure 4:**
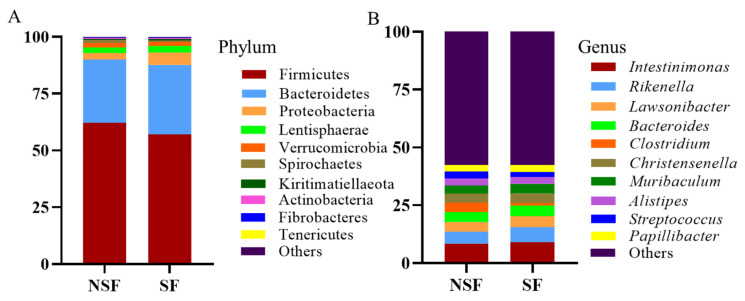
Species distribution of the gut microbiota of red deer between the two groups. (**A**) Distribution histogram of the top 10 phyla in the two groups; (**B**) distribution histogram of the top 10 genera in the two groups. The relative abundances of phyla and genera in the figure refer to the average value.

At the phylum level, the dominant phyla in both groups were Firmicutes (NSF = 62.13%, SF = 57.00%) and Bacteroidetes (NSF = 27.77%, SF = 30.66%; Figure 4A).

**Figure 5:**
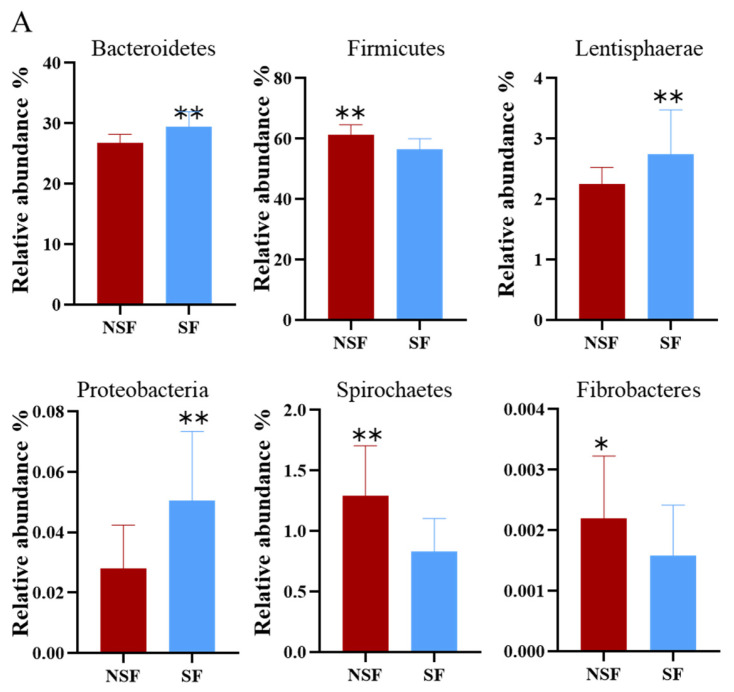

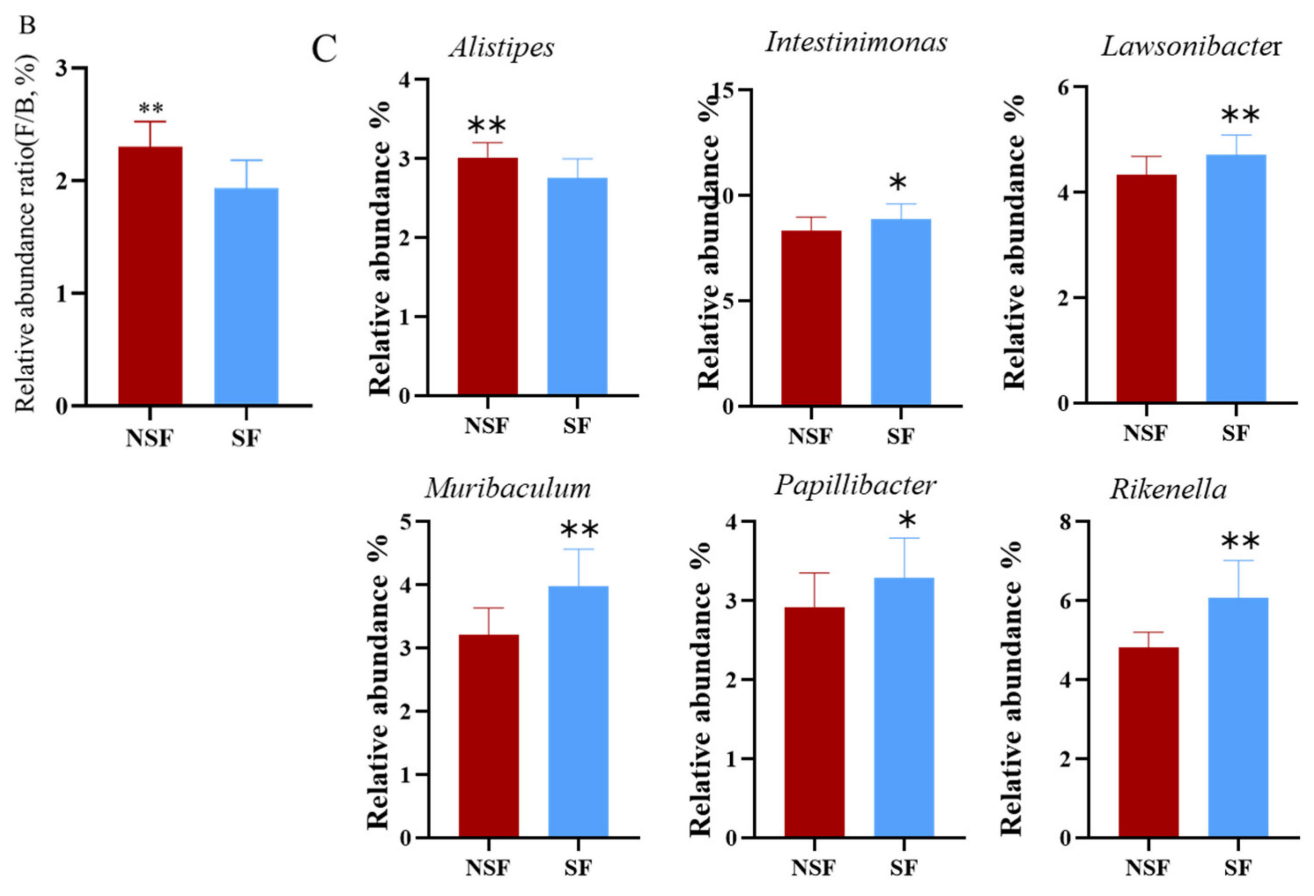
Significant differences between NSF and SF groups in the relative abundances of species. (**A**) Top 10 phyla; (**B**) Firmicutes/Bacteroidetes (F/B) ratio; (**C**) top 10 genera of red deer. Note: * *p* < 0.05, ** *p* < 0.01.

Further analysis showed that the relative abundances of Firmicutes, Spirochaetes, and Fibrobacteres in the gut microbiota of the NSF group were significantly higher than those of the SF group (Figure 5A; Firmicutes and Spirochaetes *p* < 0.01, Fibrobacteres *p* < 0.05). The relative abundance ratio of Firmicutes to Bacteroidetes (F/B) was significantly higher in the NSF group than in the SF group (Figure 5B; *p* > 0.01). However, the relative abundances of Bacteroidetes, Lentisphaerae, and Proteobacteria in the gut microbiota of the SF group were significantly higher than those of the NSF group (Figure 5A; *p* < 0.01).

Higher contributions from Firmicutes, Bacteroidetes, Proteobacteria, Lentisphaerae, Spirochaetes and Fibrobacteres were identified at the phylum level. The differences between the two groups (SF and NSF) were consistent with the above results (Table 1).

**Figure 6:**
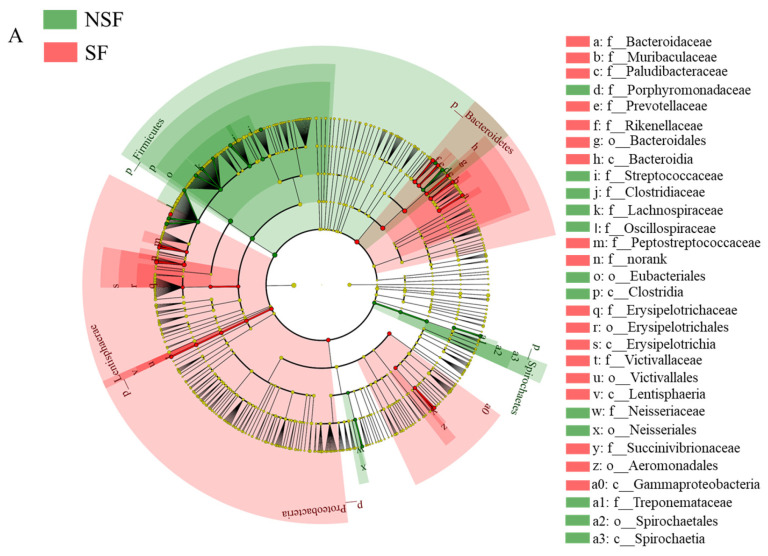

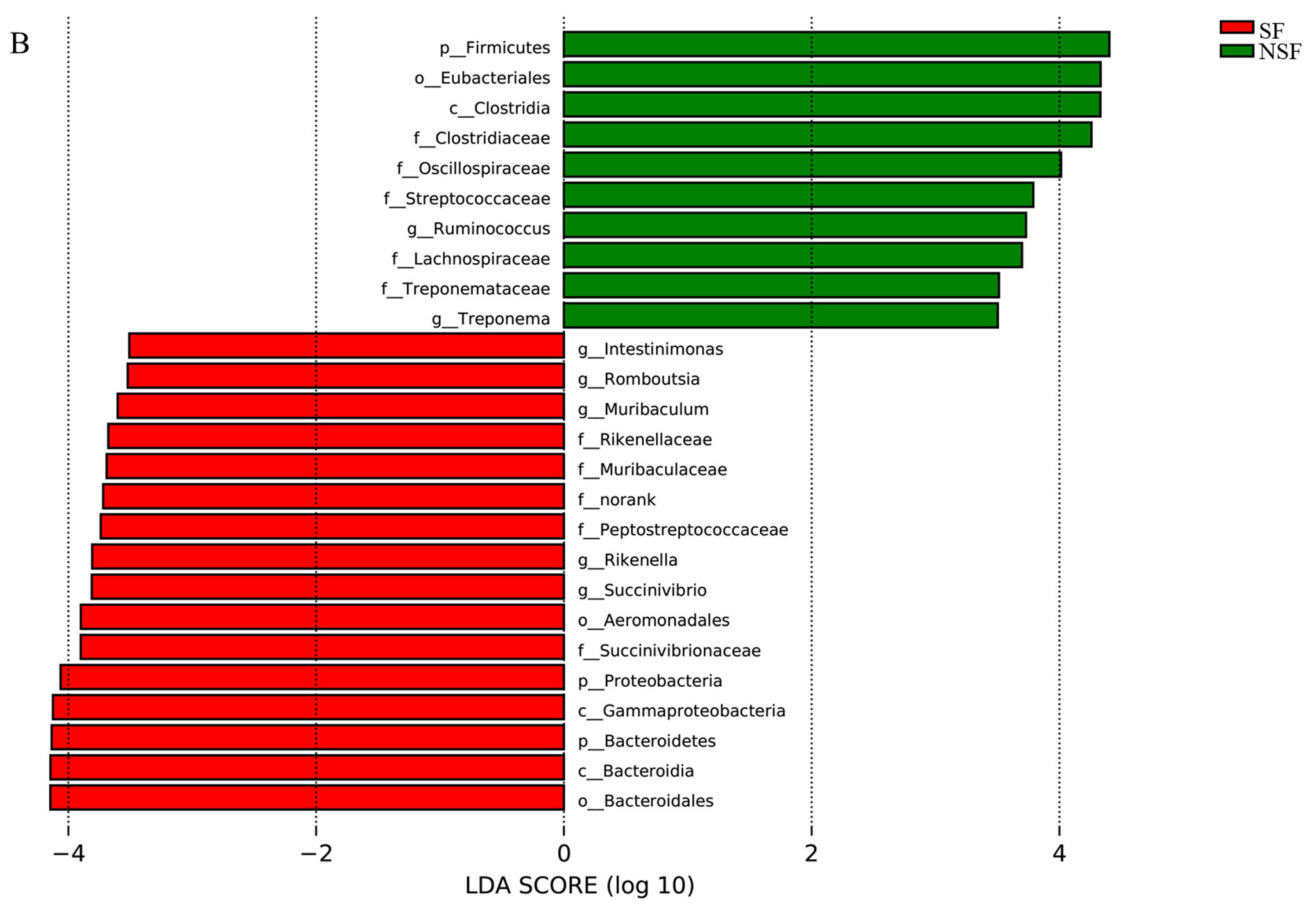
Linear discriminant analysis (LDA) effect size plots showing the taxa differing between groups of red deer. (**A**) Cladogram showing the evolutionary branching of divergent species; (**B**) distribution of LDA values for the differential biomarkers in the LDA histogram (LDA score >3.5).

*Intestinimonas* (NSF = 8.48%, SF = 9.07%) and *Rikenella* (NSF = 5.05%, SF = 6.40%) were the predominant genera in the gut microbiota of *C. elaphus* in both groups. Of the top 10 relatively abundant genera, seven showed significant differences between the two groups (Figure 5C; NSF > SF: *Alistipe*, SF > NSF: *Intestinimonas*, *Rikenella*, *Lawsonibacter*, *Muribaculum*, and *Papillibacter*). LEfSe analysis showed that biomarkers in the gut microbiota of NSF were Firmicutes, Eubacteriales, Clostridia, Clostridiaceae, and Oscillospiraceae, whereas those enriched in the gut of SF were Bacteroidales, Bacteroida, Bacteroidetes, Gammaproteobacteria, and Proteobacteria (Figure 6B, LDA > 4). At the genus level, the *Ruminococcus* and *Treponema* were the predominant genera in the gut microbiota of NSF, whereas those enriched in the gut of SF were *Succinivibrio*, *Rikenella, Muribaculum*, *Romboutsia*, and *Intestinimonas* (Figure 6B, LDA > 3.5).

The main differences in gut microbiota contributions between the two groups were centered on *Escherichia*, *Rikenella*, *Succinivibrio*, *Ruminococcus*, and *Intestinimonas*. The relative abundances of four of these genera in the SF group were significantly greater than those in the NSF group, except for *Ruminococcus* (Table 1).

### 3.4. Functional Analysis of Gut Microbiota in Red Deer

**Figure 7:**
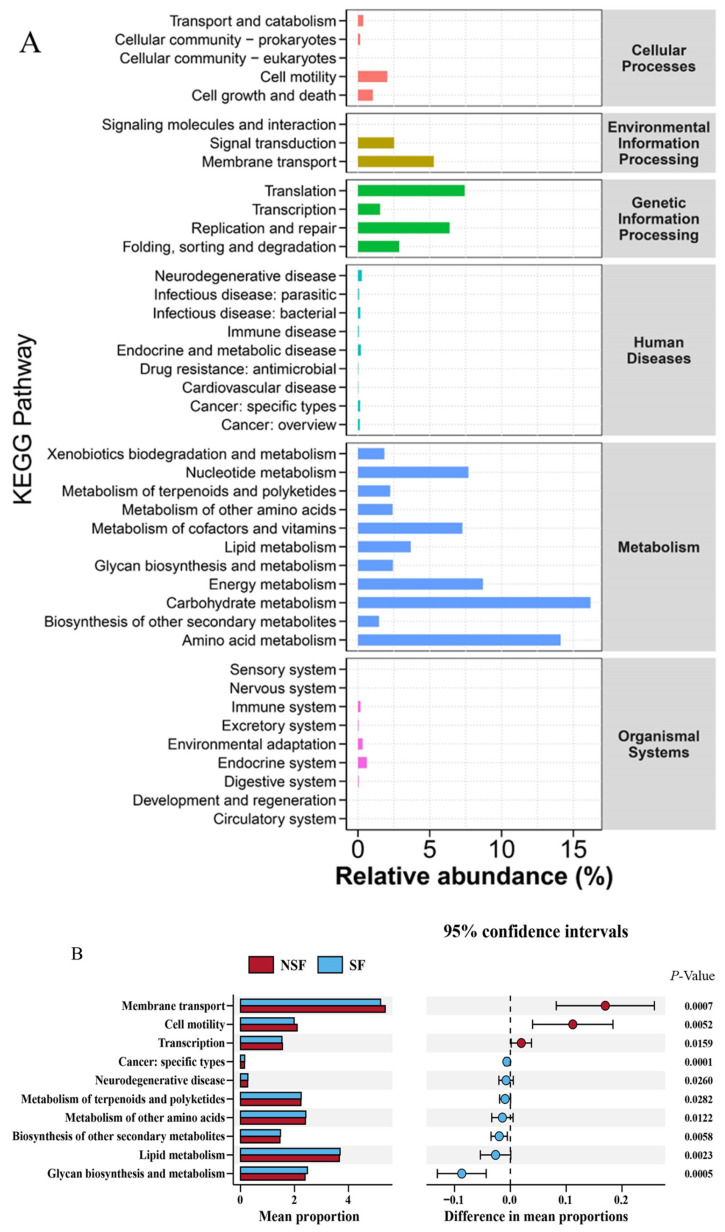
(**A**) Kyoto Encyclopedia of Genes and Genomes (KEGG) level 1 differential bubble plot for inter-group comparisons; (**B**) top 10 significant functional difference analyses of red deer gut microbiota in the KEGG level 2 pathway (*p* < 0.05).

To investigate the changes related to gut microbiota metabolism in *C. elaphus* during different groups, we used the PICRUSt2 metabolic function prediction tool based on the KEGG database. The KEGG pathways were mainly enriched in metabolism (NSF = 67.91%, SF = 68.21%; Figure 7A), including carbohydrate metabolism, amino acid metabolism, energy metabolism, nucleotide metabolism, metabolism of cofactors and vitamins, lipid metabolism, glycan biosynthesis and metabolism, metabolism of terpenoids and polyketides, metabolism of other amino acids, xenobiotics biodegradation, and metabolism and biosynthesis of other secondary metabolites (Figure 7A). Analysis of the KEGG level 2 pathways (*p* < 0.05) showed that three differentially significant pathways—membrane transport, cell motility, and transcription—were enriched in the NSF (Figure 7B), whereas, seven differentially significant pathways—cancer-specific types, neurodegenerative disease, metabolism of terpenoids and polyketides, metabolism of other amino acids, lipid metabolism, biosynthesis of other secondary metabolites, and glycan biosynthesis and metabolism—were enriched in SF (Figure 7B).

## 4. Discussion

### 4.1. Presumption 1

Studies involving captive animals showed that the alpha diversity of gut microbes is host-dependent. For example, in some mammalian hosts (such as bovids, giraffes, anteaters, and aardvarks) [53], captivity did not alter the diversity of gut microbes compared with that in wild populations. However, captivity reduced gut microbe diversity in canids, primates, and equids [53]. In contrast, in rhinoceros, gut microbe diversity increased in captivity. Overall, differences in gut microbiota diversity between captive and wild populations have been reported in most investigated species, except for Artiodactyla (bovids and giraffes) [53]. Further, other studies revealed that differences in diet do not lead to differences in the diversity and abundance of gut microbes in Père David’s deer [54]. Our results showed that the alpha diversity of the gut microbes in the NSF was not significantly different from that in the SF (Figure 2A–C). This result contrasts with Presumption 1. The gut microbiota of *C. elaphus* did not change with food in terms of diversity or with winter supplementation. We found that the main forages used for winter supplementation were maize, alfalfa, and hay, which differed in abundance from the NSF diet of *C. elaphus*. Similar to our findings, a study on the gut microbes of 22 species of large herbivorous mammals in Africa revealed that species with the most diverse diets typically did not have the most diverse microbiomes [55]. These results suggest that the effects of winter supplementation on the gut microbiota of red deer are related to their composition and function.

### 4.2. Presumption 2

At the phylum level, Firmicutes and Bacteroidetes undertake multiple metabolic roles in metabolic processes. Organisms in these phyla enhance energy extraction and improve dietary fermentation efficiency, in addition to being involved in fat accumulation for winter survival [15,56]. Interestingly, although Bacteroidetes play a prominent role during the SF phase, Firmicutes dominate during the natural grazing phase in yaks (*Bos grunniens*) [57]. Consistently, we observed Bacteroidetes abundance in the gut microbiota of *C. elaphus* in SF compared with that in NSF. This trend was also observed for Lentisphaerae and Proteobacteria. In contrast, Firmicutes, Spirochaetes, and Fibrobacteres showed the opposite abundance patterns (Figure 5A). The significant decrease in the ratio of the relative abundance of F/B (Figure 5B) was consistent with Presumption 2.

Bacteroidetes facilitate the digestion and absorption of proteins and carbohydrates in food and promote the development of the gastrointestinal immune system, whereas Firmicutes aids in fiber degradation and converts it into volatile fatty acids to support food digestion and growth [58]. In winter supplementation, high-protein feeds such as alfalfa and maize are provided, whereas during the free-range season, red deer forage on a wide variety of plants. This difference in diet may explain the greater abundance of Bacteroidetes during SF and the higher enrichment of Firmicutes and Fibrobacteres during NSF. Specifically, microorganisms enriched during NSF, as revealed by LEfSe, belonged to Firmicutes, such as Eubacteriales, Clostridia, Clostridiaceae, and Oscillospiraceae (Figure 6A,B). Eubacteriales are abundant anaerobic bacteria among gut microbiota and produce short-chain fatty acids by degrading dietary fiber [59]. Similar to our results, a previous study reported Eubacteriaceae abundance in free-ranging species such as yak, suggesting that fiber digestion and degradation during NSF are associated with Eubacteriales [57]. Similar results were obtained for Clostridiaceae. Members of the Clostridiaceae family, known for their ability to break down cellulose [60], are more prevalent in herbivores with a starch-poor diet [61]. In addition, Clostridia play a crucial role in gut homeostasis and contribute to gut health by interacting with other resident microbiota [62]. Lipid and amino acid metabolism alterations are associated with changes in Oscillospiraceae abundance [63]. Our LEfSe results indicate that Bacteroidales, Bacteroidetes, Gammaproteobacteria, and Proteobacteria were enriched in the gut microbiota associated with SF (Figure 6B). This finding agrees with that of a previous study demonstrating a higher Proteobacteria abundance in the gut microbiota of free-ranging *C. elaphus* [23].

In our study, *Intestinimonas*, *Rikenella*, and *Lawsonibacter* were the top three genera and were significantly more abundant in the SF group than in the NSF group. Previous research has indicated that *Intestinimonas* abundance significantly reduces with increases in obesity [64]. Further, *Intestinimonas* is associated with butyrate production [65] and with the biohydrogenation and utilization of volatile fatty acids in the rumen [66]. *Lawsonibacter* is also involved in the production of butyrate [67], which is associated with preventing colitis and colorectal cancer [68,69]. *Rikenella* is a crucial member of the gut microbiome and a potential probiotic that reduces intestinal inflammation [70,71]. Notably, we found that the relative abundance of *Rikenella microfusus*, *Muribaculum intestinale*, and *Escherichia coli* in the gut microbiota of red deer was significantly higher during SF than during NSF (Table S2). *R. microfusus* is an essential intestinal probiotic with great potential [72]. In previous studies in the cervids, it was found that *Escherichia coli*, *Yersinia enterolitica*, *Y. ruckerii*, *Aeromonas sobria*, *Enterococcus faecium*, *Staphylococcus aureus*, and *Lysteria monocytogenes* are potential pathogenic bacteria [23,73]. There are results showing that during the winter season, a high abundance of *E. coli* was found in the intestine of *captive equids*. [23]. Although *E. coli* is typically regarded as a potential pathogen, its average abundance was lower in both groups, suggesting that after winter supplementation, the gut microbes of red deer are dominated by probiotics, such as *R. microfusus*, *M. intestinale* [74], and *Papillibacter cinnamivorans* [75]. These gut microbes are likely involved in ameliorating obesity associated with high-energy diets. Therefore, our results indicate a shift toward gut microbes associated with improved metabolic health after winter supplementation, which contrasts with Presumption 2.

### 4.3. Presumption 3

The KEEG results showed that the pathways with a higher percentage of significant differences in SF were associated with lipid metabolism, glycan biosynthesis and metabolism, and the metabolism of other amino acids (Figure 7B). In ruminant-related studies, it was found that a high-energy diet increased lipid metabolism in the microbiota. Carbohydrate-activating enzyme (CAZy) genes involved in energy metabolism were upregulated, while genes regulating plant cell wall degradation were downregulated in the high-energy group [76]. This is similar to our findings that microbial involvement in lipid metabolism was significantly greater after high-energy supplementation than in the NSF.

Red deer require sufficient fat and energy to cope with the severe winter climate. In supplemental feeding recipes, alfalfa and maize fulfill the protein and energy requirements of the animals. Abundances of gut microbes such as *Intestinimonas* and *Rikenella* increase during SF-mediated lipid metabolism and intestinal homeostasis, which are crucial when *C. elaphus* consumes high-energy foods [64,77]. Specifically, species (Figure S2) such as *I. butyriciproducens*, *I. massiliensis*, and *I. timonensis* are associated with butyrate production in the gut microbes of *C. elaphus* during SF [78,79,80], which is an energy source for epithelial cells and plays a key role in colonic cell homeostasis maintenance. Notably, butyrate exerts an inhibitory effect on inflammation and oxidative stress [81]. Further, like other SCFAs, butyrate contributes to the improvement of insulin sensitivity and glucose homeostasis [82]. Genome analysis predicted that *I. timonensis* can utilize starch, sulfide, and L-serine to produce acetate, butyrate, propionate, L-cysteine, and riboflavin (vitamin B2) [80,83]. Interestingly, the higher starch content in maize diets at KEGG level 3 also predicted significantly greater riboflavin metabolism during SF compared to during NSF (Figure S3). These results support Presumption 3, further indicating that feeding enrichment alters the gut microbiota composition in captive *C. elaphus*. In the absence of dietary data from the NSF, verifying the link between diet and gut microbiology in the NSF of the red deer was not possible, and this should be further evaluated.

## 5. Conclusions

No significant differences were found in the diversity of the red deer gut microbiota between the two groups (SF and NSF) except for the Simpson’s index. Instead, there were significant differences in composition and function, with the SF group enriched in Bacteroidetes, Lentisphaerae, and Proteobacteria, and at the genus level predominantly enriched in *Intestinimonas*, *Rikenella*, *Lawsonibacter*, *Muribaculum*, and *Papillibacter*. Microbes in the SF were primarily associated with lipid and butyrate metabolism, whereas microbes in the NSF were associated with fiber catabolism. Future research is needed to evaluate the effects of dietary studies on the gut microbiology of wild red deer, providing new perspectives on captive breeding. Overall, supplemental feeding provided the necessary nutrients for red deer during the winter and increased the abundance of probiotics in their intestinal tract, demonstrating that winter supplemental feeding is a reasonable and effective management strategy.

## Figures and Tables

**Table 1:** Analysis of differential gut microbiota contribution of red deer between non-supplementary feeding season and supplementary feeding season.

**Phylum**	**Average**	**SD**	**Ratio**	**ava**	**avb**	**cumsum**	***p***
Firmicutes	0.028	0.020	1.435	62.132	57.000	0.365	0.001
Bacteroidetes	0.016	0.012	1.292	27.769	30.659	0.566	0.001
Proteobacteria	0.014	0.011	1.297	2.952	5.313	0.752	0.002
Verrucomicrobia	0.008	0.008	1.118	2.040	1.770	0.861	0.212
Lentisphaerae	0.003	0.003	0.990	2.346	2.872	0.904	0.004
Spirochaetes	0.003	0.002	1.231	1.355	0.871	0.939	0.001
Actinobacteria	0.002	0.003	0.637	0.268	0.448	0.965	0.173
Kiritimatiellaeota	0.001	0.001	1.375	0.511	0.524	0.977	0.429
Fibrobacteres	0.001	0.000	1.440	0.230	0.166	0.984	0.046
Tenericutes	0.000	0.000	1.478	0.150	0.167	0.988	0.173
**Genus**	**Average**	**SD**	**Ratio**	**ava**	**avb**	**cumsum**	***p***
*Clostridium*	0.020	0.024	0.835	4.053	1.138	0.091	0.081
*Streptococcus*	0.012	0.009	1.348	3.033	2.111	0.146	0.218
*Akkermansia*	0.008	0.008	1.083	1.975	1.616	0.184	0.24
*Escherichia*	0.008	0.012	0.658	0.432	1.622	0.220	0.011
*Rikenella*	0.007	0.004	1.634	5.053	6.400	0.252	0.001
*Succinivibrio*	0.006	0.009	0.742	0.006	1.276	0.281	0.001
*Lactobacillus*	0.006	0.010	0.577	1.549	1.411	0.308	0.227
*Ruminococcus*	0.006	0.003	2.233	2.078	0.926	0.334	0.001
*Intestinimonas*	0.005	0.003	1.400	8.486	9.076	0.355	0.028

(Note: ava: mean abundance of differential gut microbiota in the NSF; avb: mean abundance of differential gut microbiota in the SF; cumsum: cumulative proportions of between-group difference contributions).

## Data Availability

Data are contained within the article and Supplementary Materials. The raw sequencing data have been deposited in the NCBI Sequence Read Archive (SRA) within the BioProject PRJNA1088782.

## Supplementary Materials

The following supporting information can be downloaded at: https://www.mdpi.com/article/10.3390/ani14101428/s1. Table S1: Composition of the diets during in winter; Table S2: Analysis of differential gut microbiota (Species) contribution between non-supplementary feeding season and supplementary feeding season; Figure S1: Percentage of the phyla in core microbiome; Figure S2: Significant differences between groups (NSF: non-supplementary feeding season; SF: supplementary feeding season) of relative abundance of species in Top 10 species; Figure S3: The significant functional difference analyses of red deer gut microbiota in the KEGG level 3 pathway (NSF: non-supplementary feeding season; SF: supplementary feeding season, *p* < 0.05).
