# Supplementary material for: High-Energy Supplemental Feeding Shifts Gut Microbiota Composition and Function in Red Deer (Cervus elaphus)
Source: Animals (Basel). 2024 May 10;14(10):1428. doi: 10.3390/ani14101428 (PMC11117297; doi:10.3390/ani14101428)
Supplement: Supplementary file 1 [file animals-14-01428-s001.zip › animals-2978969-supplementary.pdf]

Table S1 Composition of the diets during in winter

Item	Mass (kg)
corn	68
bran (esp. of wheat)	15
Sunflower seed	10
Bentonite	1.5
calcium bicarbonate	2
salt	1
trace element	1
Alfalfa Grass Powder	1.5

The figures in the table are the weight of each nutrient species per 100 kg, except for 75 kg of water per 100 Kg.

Table S2 Analysis of differential gut microbiota (Species) contribution between non-supplementary feeding season and supplementary feeding season.

Species	average	sd	ratio	ava	avb	cumsum	*P*
Clostridium sp000753455"	0.01969	0.024935	0.789651	3.889571	0.639024	0.077175	0.065
Streptococcus thermophilus	0.009335	0.006978	1.337773	2.393718	1.256996	0.113763	0.071
Akkermansia muciniphila	0.008248	0.009228	0.893852	1.597044	0.612222	0.146092	0.117
Escherichia flexneri	0.007724	0.011842	0.652255	0.39971	1.588175	0.176366	0.016
Rikenella microfusus	0.006472	0.003904	1.657727	4.608547	5.820269	0.201734	0.001
Succinivibrio dextrinosolvens_A	0.005476	0.007423	0.737699	0.005128	1.099795	0.223197	0.001
Unclassified	0.004983	0.00366	1.361566	9.849822	10.39452	0.242727	0.007
Muribaculum intestinale	0.004389	0.002955	1.485221	3.315796	4.102524	0.259929	0.001
Lactobacillus crispatus	0.003668	0.008883	0.412861	0.387721	0.429339	0.274305	0.551
Proteiniphilum sp012837345	0.00348	0.003182	1.093377	1.387824	1.35286	0.287942	0.694

Note: ava: Mean abundance of differential gut microbiota in the NSF: avb: Mean abundance of differential gut microbiota in the SF cumsum: Proportion of cumulative between-group differences contributing

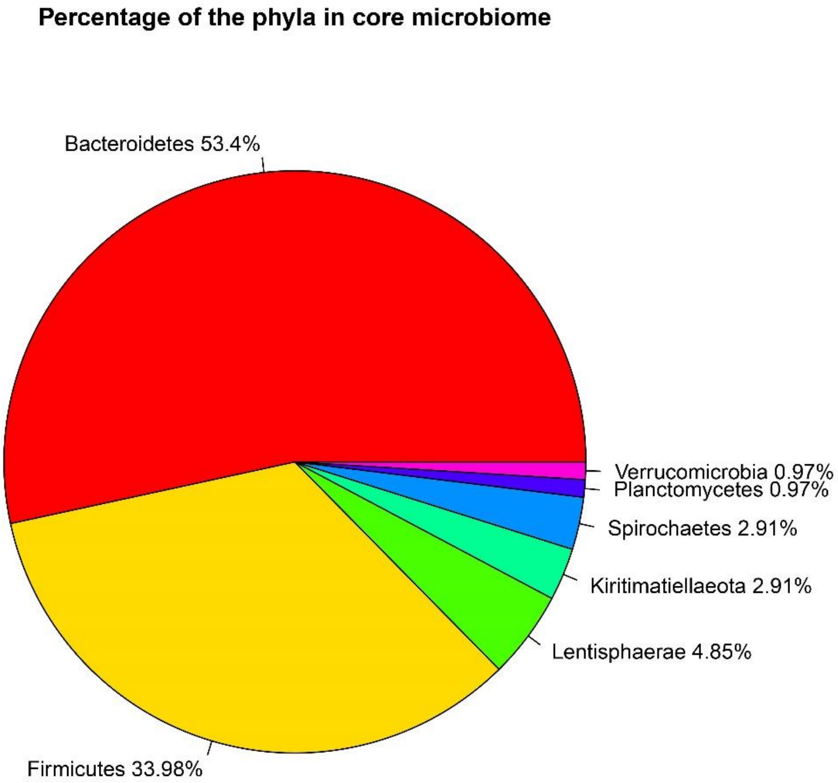

Figure S1 Percentage of the phyla in core microbiome

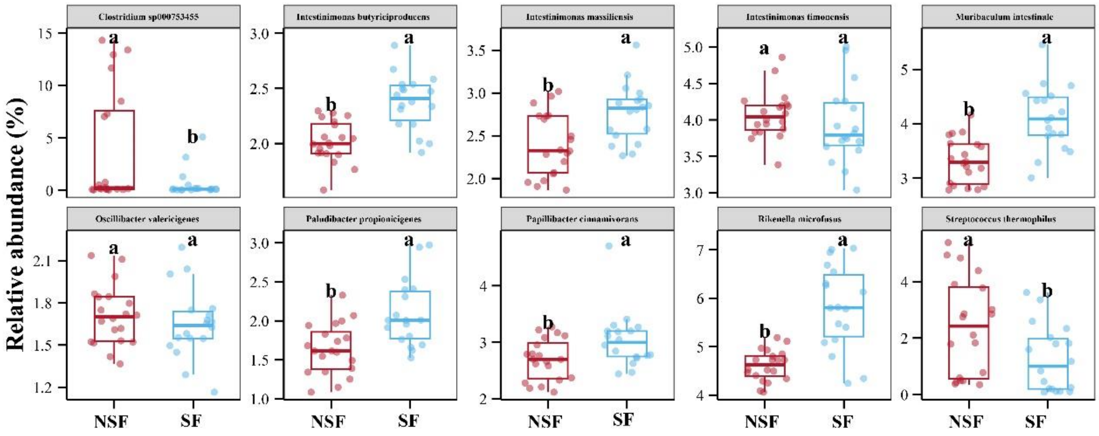

Figure S2 Significant differences between groups (NSF: non-supplementary feeding season; SF: supplementary feeding season) of relative abundance of species in Top10 species.

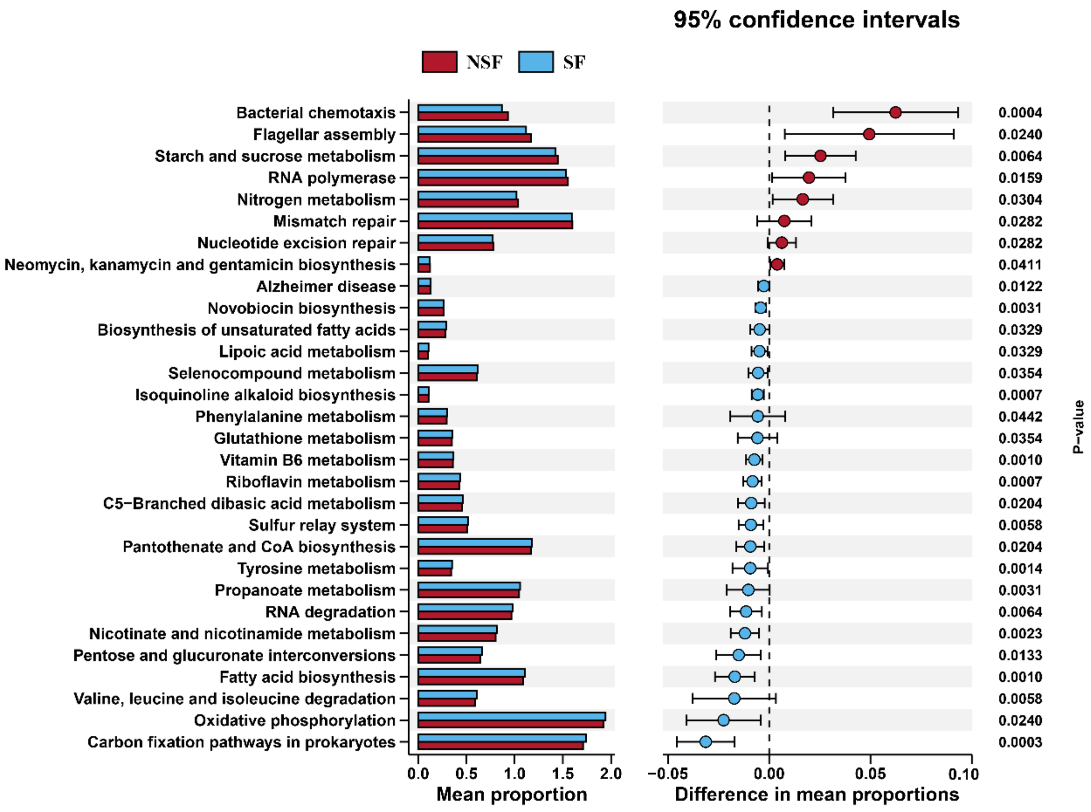

Figure S3. The significant functional difference analyses of red deer gut microbiota in the KEGG level 3 pathway (NSF: non-supplementary feeding season; SF: supplementary feeding season, $p < 0.05$)
